# HCK promotes glioblastoma progression by TGFβ signaling

**DOI:** 10.1042/BSR20200975

**Published:** 2020-06-17

**Authors:** Zhenlin Wang, Chenting Ying, Anke Zhang, Houshi Xu, Yang Jiang, Meiqing Lou

**Affiliations:** 1 Department of Neurosurgery, Shanghai General Hospital, Shanghai Jiao Tong University School of Medicine, 100 Haining Road, Shanghai, China; 2 Department of Orthopedics, Shanghai General Hospital, Shanghai Jiao Tong University School of Medicine, 100 Haining Road, Shanghai, China

**Keywords:** epithelial mesenchymal transition, glioblastoma, HCK, N-cadherin, Smad2/3

## Abstract

The hematopoietic cell kinase (HCK), a member of the Src family protein-tyrosine kinases (SFKs), is primarily expressed in cells of the myeloid and B lymphocyte lineages. Nevertheless, the roles of HCK in glioblastoma (GBM) remain to be examined. Thus, we aimed to investigate the effects of HCK on GBM development both *in vitro* and *in vivo*, as well as the underlying mechanism. The present study found that HCK was highly expressed in both tumor tissues from patients with GBM and cancer cell lines. HCK enhanced cell viability, proliferation, and migration, and induced cell apoptosis *in vitro*. Tumor xenografts results also demonstrated that HCK knockdown significantly inhibited tumor growth. Interestingly, gene set enrichment analysis (GSEA) showed HCK was closed associated with epithelial mesenchymal transition (EMT) and TGFβ signaling in GBM. In addition, we also found that HCK accentuates TGFβ-induced EMT, suggesting silencing HCK inhibited EMT through the inactivation of Smad signaling pathway. In conclusion, our findings indicated that HCK is involved in GBM progression via mediating EMT process, and may be served as a promising therapeutic target for GBM.

## Introduction

Gliomas, the most common primary brain tumor in adults, included astrocytoma, oligodendroglioma, mixed glioma, medulloblastoma, and ependymoma [1]. Glioblastoma (GBM) belongs to astrocytoma, and is classified as grade IV by World Health Organization (WHO) [2]. The standard therapies for GBM involve maximal surgical resection, followed by radiotherapy and alkylating chemotherapy with temozolomide [3]. In the recent years, its overall prognosis is poor despite the aggressive standard therapies [4]. The median survival of optimally treated GBM patients is 14 months, with a 4–5% 5-year survival rate [5–7].

The hematopoietic cell kinase (HCK), a non-receptor or cytoplasmic tyrosine kinase, belongs to the Src family protein-tyrosine kinases (SFKs), which regulate a series of cellular processes, including mitogenesis, differentiation, survival, migration, and adhesion [8]. The expression of HCK is limited to the hematopoietic system, primarily including cells of the myeloid and B lymphocyte lineages [9,10]. Excessive activation of HCK is reported to be associated with various leukemia, such as chronic myeloid leukemia (CML), multiple myeloma, and acute lymphoblastic leukemia, as well as solid malignancies including colorectal, breast, and gastric cancer [11–13]. In CML, HCK is activated by BCR/ABL, an oncogenic fusion protein in a large majority of CML as well as in some acute lymphocytic leukemia, leading to the persistent activation of STAT5 and its excessive accumulation in the cytoplasm, where STAT5 activates AKT through combining with PI3K and the adaptor protein GAB2, promoting cell growth and survival [14,15]. HCK is also identified as an intermediate member of the erythropoietin/erythropoietin receptor and PI3K/AKT or MAPK/ERK pathway, playing a critical role in GATA-1 and BCL-XL modulation, erythropoiesis maturation, and cell death [16]. In cancer, HCK activation interacts with receptor tyrosine kinases (RTK), such as platelet-derived growth factor receptor (PGDFR), epidermal growth factor receptor (EGFR), and fibroblast growth factor receptor (FGFR), activating ERK, AKT, and STAT3 signaling pathways, further stimulating cell proliferation [14]. In addition, HCK plays a role in chemo resistance [17–19] and reduced drug efficacy in clinical study [20].

HCK activation may indirectly promoting tumor progression through cytokine secretion in tumor-related immune cells, except its direct oncogenic role in cancer cells. It plays an important role in the innate immune response through modulating the proliferation and migration of neutrophil phagocytosis and macrophage [12]. Furthermore, HCK is also associated with inflammatory signaling. It is involved in CD14/Toll-like receptor (TLR) 4 cell signaling, and regulates the production of TNF-a, IL-1, and IL-6 stimulated by lipopolysaccharide, resulting in inflammation perpetuation and neoplastic transformation sustentation in tumor cell [13,14].

However, the function of HCK in GBM remains to be examined. The present study found that HCK was highly expressed in both tumor tissues from patients with GBM and cancer cell lines. Inhibition of HCK suppressed GBM development both *in vitro* and *in vivo*. Moreover, gene set enrichment analysis (GSEA) analysis showed HCK was closely associated with EMT and TGFβ signaling, and we also discovered a positive correlation between HCK and N-cadherin, but an inverse relationship between HCK and E-cadherin. These findings suggest that HCK is involved in GBM progression via mediating EMT process, and may be served as a promising therapeutic target for GBM.

## Materials and methods

### TCGA cancer analysis and gene set enrichment analysis

HCK expression between tumor and normal tissues of GBM were analyzed. The sample data from 163 tumor tissues and 207 normal tissues were obtained from the TCGA database. To reveal the function of HCK in GBM, GSEA was performed.

### Cell culture and expression constructs

Three GBM cell lines (U251, SHG-44, and SNB-19 cells) were obtained from American Type Culture Collection (ATCC; Manassas, U.S.A.), and one normal glial cell (HEB cell) was purchased from Beijing Beina Biotechnology Academy (Beijing, China) All cells were cultured in Dulbecco's Modified Eagle's Medium (DMEM; Invitrogen, Carlsbad, CA, U.S.A.) supplied with 10% fetal bovine serum (FBS; HyClone, South Logan, UT, U.S.A.) at 37°C in an atmosphere with 5% CO_2_. The pCDNA-Flag-hHCK plasmid was constructed in the present study. The primer of HCK were designed in accordance with pcDNA3.1 vector sequence and synthetized by Shanghai BioSune Biological Technology Co., Ltd. The HCK sequences with double cleavage sites were amplified by PCR and connected to the pcDNA3.1 vector after double enzyme cutting. The plasmid that expressed HCK successfully were screened and identified. All the constructs were verified by DNA sequencing.

### Quantitative reverse transcription polymerase chain reaction (qRT-PCR)

Total RNA was extracted from HEB, U251, SHG-44, and SNB-19 cells using TRIzol reagent (TaKaRa Bio, Kusatsu, Japan). After RNA concentration detection, cDNA was synthesized from total RNA using First-Strand cDNA Synthesis kit (TaKaRa Bio, Kusatsu, Japan). Next, SYBR was used for PCR amplification by StepOnePlus Real-Time PCR System (Applied Biosystems). The sequences of the primers were listed below. HCK, F: 5′-CTGCCAACATCTTGGTCTCT-3′; R: 5′- TCAGCAGGATACCAAAGGAC-3′.

### Cell transfection

To construct stable cell lines with HCK knockdown, U251 and SHG-44 cells were transfected with HCK siRNA or lentiviral shRNA vector. Cell were seeded in six-well plates for one day. After 40–50% confluence, siRNA or lentiviral shRNA vector was transfected along with Lipofectamine 2000 into GBM cells according to supplied protocol. And cells were transferred to complete medium 6 h after transfection. The levels of HCK was evaluated by western blotting assay. The siRNA against HCK was purchased from MERCK (Germany). The shRNA against HCK shown as follows: CCGGTACCAACAGCAGCCATGATAGCTCGAGCTATCATGGCTGCTGTTGGTATTTTTG.

### Western blotting assay

Total proteins from cells were extracted using lysis buffer, and protein concentration was measured. Next, proteins were separated by 10% sodium dodecyl sulphate/polyacrylamide gel electrophoresis (SDS/PAGE), and further transferred to polyvinylidene difluoride (PVDF) membranes, which were then blocked by non-fat milk for 2 h at room temperature. After blockade, the membrane was incubated with primary antibodies at 4°C overnight, followed by incubation with HRP-conjugated anti-rabbit secondary antibodies at 4°C for 2 h. In the end, the membranes were visualized using enhanced chemiluminescence reagents.

### Cell Counting Kit (CCK)-8 assay

Cell Counting Kit (CCK)-8 assay was used to measure cell viability. After transfection, cells were collected and plated in 24-well plates at 37°C for 24, 48, 72, and 96 h. CCK-8 reagent (Sigma–Aldrich) was supplemented into each well, and incubated with cells for 2 h. And then, the absorbance was measured at 450 nm using a microplate reader (Thermo Fisher Scientific, Waltham, MA, U.S.A.).

### Colony formation assay

After transfection, cells were collected and plated in six-well plates at 37°C for 2–3 weeks. Next, cells were fixed with 4% paraformaldehyde for 30 min, and stained with crystal violet for 30 min. The number of colonies in each well were counted.

### Transwell assay

The ability of cell migration was measured using transwell assay. After transfection, cells were seeded in the upper chamber (8 µm) coated with 100 µl Matrigel (BD Biosciences, CA, U.S.A.). The lower chamber was filled with DMEM supplemented with 20% FBS. The chambers were incubated at 37°C with 5% CO_2_ for 24 h. Next, cells in the lower chamber were fixed by 4% paraformaldehyde, and stained by crystal violet. The number of stained cells was counted under an inverted microscope.

### Caspase-3 activity assay

To evaluate cell apoptosis, caspase-3 activity assay kit (Beyotime) was used according to the manufacturer's instructions. After transfection, cells were collected, and lysed with lysis buffer on ice for 15 min. Caspase-3 substrate were added into cell lysate, and incubated at 37°C for 4 h. In the end, caspase-3 activity was detected.

### Xenograft experiments

The animal work has taken place in Shanghai Jiao Tong University School of Medicine, and the ethics approval has been obtained from Shanghai Jiao Tong University School Institutional Animal Care and Use Committee, No. 20200315. After transfection with shRNA, U251 cells were subcutaneously injected into the right of the center in the back of Balb/c nude mice at the age of 4–5 weeks. Tumor volume was measured. After 21 days, the mice were killed by i.p. injection of 1 ml of 2.5% avertin per mouse.

### Statistical analysis

All data were analyzed using SPSS 21.0 software, and showed as means ± standard deviation (SD) from three independent experiments. Student's *t*-test and one way analysis of variance (ANOVA) were used to analyze the difference between two groups and among multiple groups. *P*<0.05 was considered as significant difference in the present study.

## Results

### HCK was up-regulated in GBM tissues and GBM cell lines

**Figure 1:**
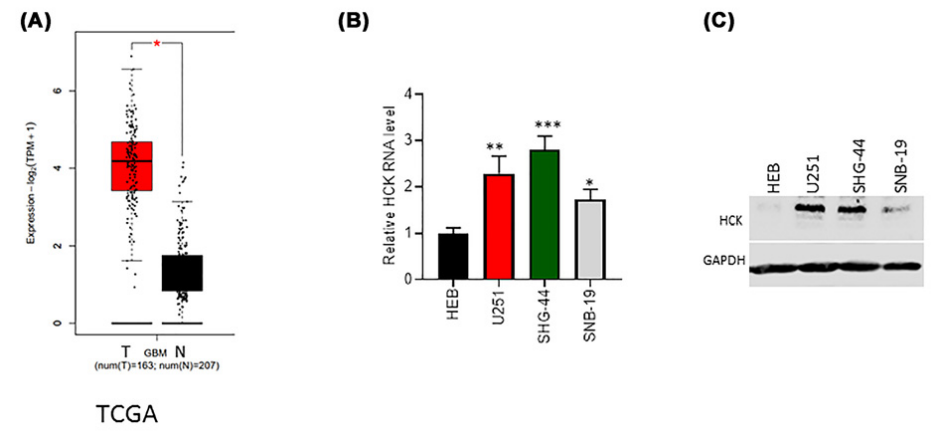
HCK was highly expressed in both tumor tissues from patients with GBM and GBM cell lines (**A**) The expression levels of HCK in 163 tumor tissues and 207 normal tissues were analyzed by TCGA. The RNA (**B**) and protein levels (**C**) of HCK in GBM cancer lines (U251, SHG-44, and SNB-19 cells) and normal glial cell (HEB cell). *P<0.05. **P<0.01. ***P<0.001.

To evaluate the expression levels of HCK in GBM, we first analyzed TCGA databases. A total of 163 tumor samples from GBM patients and 207 normal samples were analyzed, and we found that HCK was highly expressed in tumor samples compared with control samples (Figure 1A). Next, we investigated HCK expression in GBM cell lines (U251, SHG-44, and SNB-19 cells), and normal glial cells (HEB cell). The results showed that the RNA and protein levels of HCK were both significantly increased in GBM cells compared with HEB cell (Figure 1B,C). However, HCK was weakly expressed in SNB-19 in relative to the other two GBM cells. Thus, we used U251 and SHG-44 cells for the follow-up studies.

### Inhibition of HCK reduced cell proliferation in GBM cell lines

**Figure 2:**
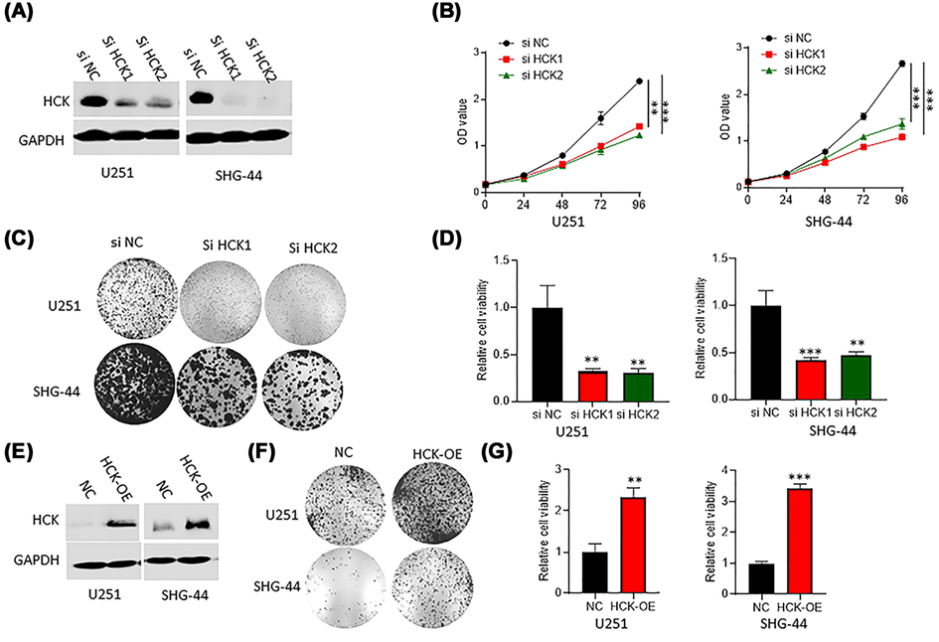
Inhibition of HCK suppressed cell viability and proliferation in U251 and SHG-44 cells (**A**) Western blotting assay verified HCK expression in cells transfected with HCK siRNA. (**B**) CCK-8 assay detected the role of HCK knockdown on cell viability. (**C,D**) Colony formation assay examined the effect of HCK knockdown in cell proliferation. **P<0.01. ***P<0.001. (**E**) Western blotting assay tested HCK's expression in HCK-OE U251 or SHG-44 cells. (**F,G**) Colony formation assay examined the effect of HCK overexpression in cell proliferation. **P<0.01. ***P<0.001.

To investigate the effects of HCK on GBM, gain-of-function and loss-of-function methods were used. SiHCK assay was carried out to knock down HCK in GBM cell lines (U251 or SHG-44). After transfection, HCK expression was detected by western blotting assay (Figure 2A). In addition, CCK-8 assay and colony formation assay were performed to test cell viability and proliferation. The results of CCK-8 assay showed a weaker viability of cells in GBM cells with HCK inhibition than that in the control group (Figure 2B). The colony formation assay showed a same result that HCK inhibition caused a significant decline of cell proliferation in U251 and SHG-44 cells (Figure 2C,D). Next, we generated the HCK overexpressed (HCK-OE) U251 or SHG-44 cells (Figure 2E). We defined that overexpression of HCK significantly promoted the growth of U251 or SHG-44 cells (Figure 2F,G).

### HCK promotes GBM cell migration and inhibits cell apoptosis

**Figure 3:**
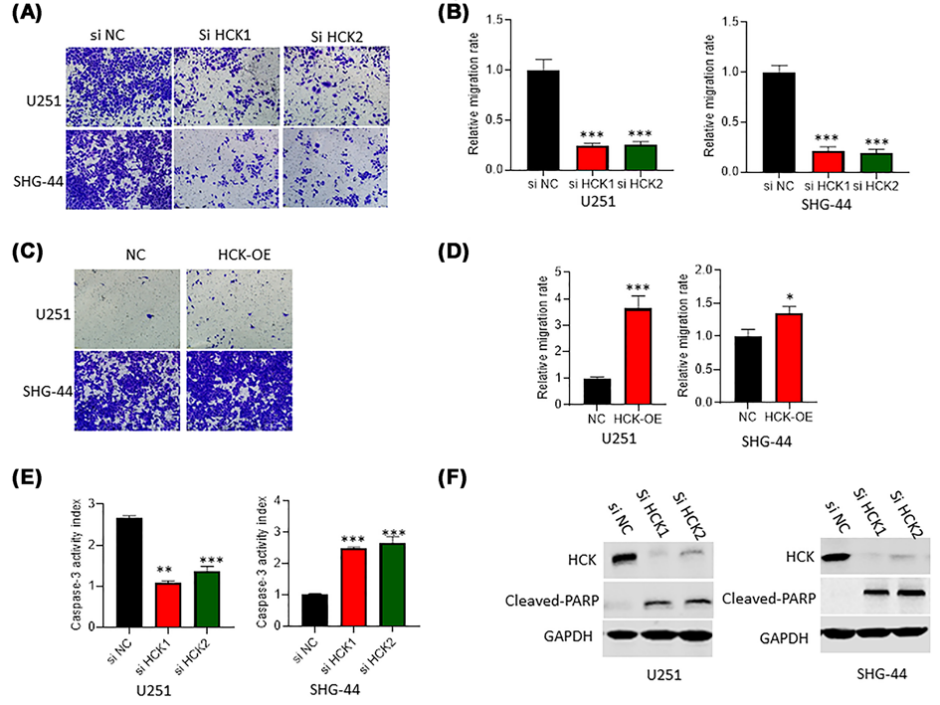
Inhibition of HCK reduced cell migration and increased cell apoptosis (**A,B**) The role of HCK knockdown in cell migration was determined by transwell assay. (**C,D**) Transwell assays experiments were performed and statistical analysis was shown in HCK-OE U251 or SHG-44 cells. *P<0.05. ***P<0.001. (**E**) Caspase-3 activity was promoted caused by HCK knockdown. (**F**) The protein levels of cleaved-PAPR were increased in cells with HCK knockdown compared with control cells. **P<0.01. ***P<0.001.

Using transwell matrigel assays to measure migration and invasion, we found higher metastatic potential in shN cells compared with HCK knockdown U251 and SHG-44 cells (Figure 3A,B). Conversely, overexpressed HCK significantly displayed increased invasion/migration capabilities (Figure 3C,D). Caspase-3 activity assay showed that HCK knockdown significantly increased caspase-3 activity in GBM cells compared with control cells (Figure 3E). Furthermore, the results of western blotting assay revealed that the protein levels of cleaved-PARP were higher in GBM cells with HCK inhibition compared with that in control cells (Figure 3F). Caspase-3 and cleaved PARP are both biomarkers of cell apoptosis, and cleaved PARP is the hydrolysis products of caspase-3. Thus, these findings suggest that HCK knockdown promoted cell apoptosis.

### HCK accentuates TGFβ-induced EMT

**Figure 4:**
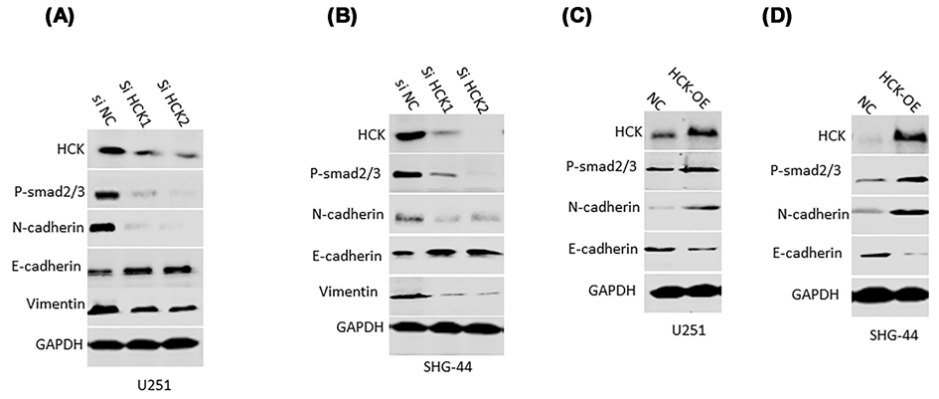
HCK promoted the expression of P-Smad2/3 and N-cadherin and inhibited the expression of E-cadherin The protein levels of P-Smad2/3, N-cadherin, E-cadherin, and vimentin of HCK knockdown U251 (**A**) and SHG-44 (**B**) cells were measured by western blotting assay. The expression of P-Smad2/3, N-cadherin, E-cadherin, and vimentin was examined by western blotting in HCK overexpressed U251 (**C**) and SHG-44 (**D**) cells.

In the present study, we performed GSEA and found several pathways involved in GBM development. Among these pathways, HCK was closely associated with epithelial mesenchymal transition (EMT), hypoxia, and TGFβ signaling (Supplementary Figure S1). TGFβ and hypoxia have been reported to trigger the process of EMT [21,22]. Thus, to verify the association between HCK and EMT, we detected the expression of Smad2/3, an intracellular signaling molecule activating different EMT transcription factors, and N-cadherin, an EMT marker. Western blotting assay demonstrated a decline of P-Smad2/3 and N-cadherin protein levels, but a negative association of HCK with E-cadherin in HCK knockdown U251 and SHG-44 cells, suggesting HCK might be involved in GBM via EMT (Figure 4A,B). Intriguingly, we discovered a positive correlation between HCK and N-cadherin, but an inverse relationship between HCK and E-cadherin (Figure 4C,D) in HCK overexpressed U251 and SHG-44 cells. We validated our observations that HCK accentuates TGFβ-induced EMT.

### Inhibition of HCK attenuated tumor growth *in vivo*

**Figure 5:**
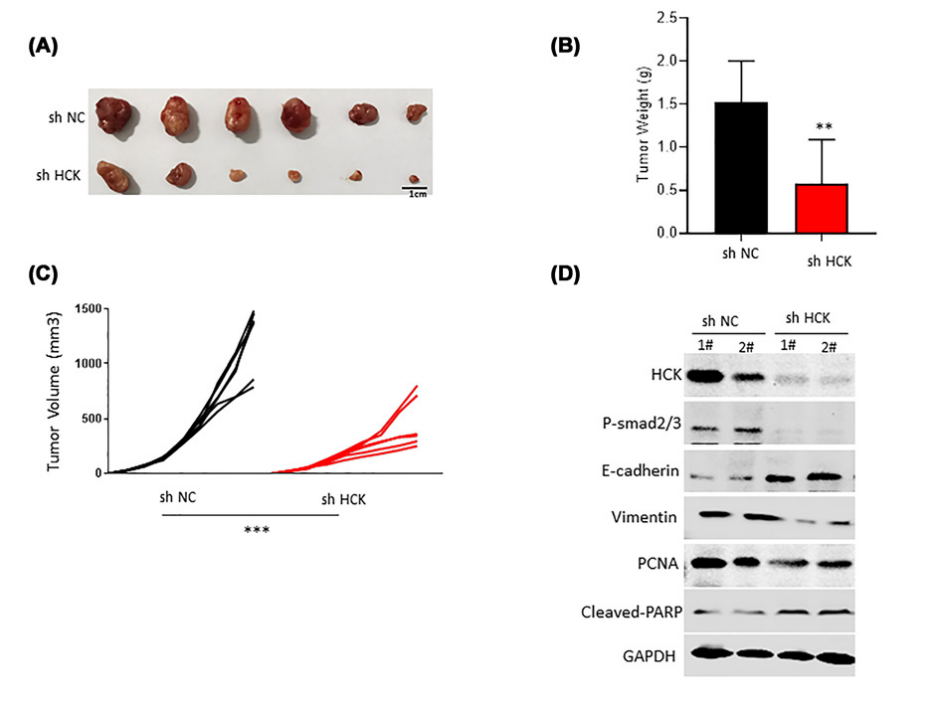
HCK knockdown inhibited tumor growth *in vivo* (**A**) The images of tumors in nude mice bearing xenograft U251 cells. (**B**) Tumor weight was detected. **P<0.01. (**C**) Tumor volume was detected every three days. ***P<0.001. (**D**) The protein expression of HCK, P-Smad2/3, E-cadherin, PCNA, and cleaved-PARP levels was analyzed by Western blotting.

To gain additional insights into the function of HCK *in vivo*, stable HCK silenced U251 cells (sh HCK) were generated. sh HCK or control U251 cells were injected into nude mice and the tumor was observed. The results showed that the tumor size was smaller in mice with HCK inhibition than that in control mice (Figure 5A). Tumor weight and tumor volume were also decreased in mice with HCK compared with that in control mice (Figure 5B,C). Furthermore, we detected the HCK, P-Smad2/3, E-cadherin, PCNA (a proliferation marker), and cleaved-PARP levels in tumors derived from tumors and found the expression of HCK still decreased in the dissected tumors (Figure 5D). Comparative analysis of sh HCK and control tissues showed a negative correlation between HCK and E-cadherin and decreased P-smad2/3, vimentin, and PCNA levels were also observed in sh HCK tumor samples (Figure 5D). These findings suggest that knockdown of HCK significantly inhibited tumor growth.

## Discussion

The HCK, a member of SFKs, is expressed in hematopoietic system, primarily including cells of the myeloid and B lymphocyte lineages [9,10]. Excessive activation of HCK is associated with various leukemia, such as CML, multiple myeloma, and acute lymphoblastic leukemia, as well as solid malignancies including colorectal, breast, and gastric cancer [11–13]. Nevertheless, the roles of HCK in GBM remain to be examined. Therefore, the present study is intended for the exploration of how HCK functions in GBM progression by using cell experiment *in vitro* and mice trial *in vivo*. Our results revealed that inhibition of HCK led to the decrease in cell viability, migration, and tumor growth, as well as the increase in cell apoptosis via inhibiting EMT.

In the present study, HCK was demonstrated to be highly expressed in both tumor tissues from patients with GBM and GBM cell lines. Previous studies also reported excessive HCK expression in colorectal, breast, and gastric cancer [11–13]. Subsequently, we analyzed the effects of HCK on GBM development both *in vitro* and *in vivo*. In colorectal cancer, HCK is involved in decreased proliferation and poorer differentiation, suggesting HCK overexpression promotes tumorigenesis [12]. Likewise, in mice excessive HCK activity promotes the growth of endogenous colonic malignancies and of human colorectal cancer cell xenografts [10]. In the present study, inhibition of HCK caused a decline of cell viability, proliferation, and migration, and induced cell apoptosis. The same result was observed in mice xenograft model. Decreased HCK activity inhibited tumor growth. These findings suggest that HCK may be served as a promising therapeutic target for GBM.

The mechanism by which HCK involved in GBM development was investigated. Our GSEA showed that HCK was closely associated with EMT, hypoxia, and TGFβ signaling. TGFβ and hypoxia have been reported to trigger the process of EMT, suggesting HCK may play an important role in EMT in GBM development [22,23]. EMT, a trans differentiation process converting epithelial cells into motile mesenchymal cells, is involved in the induction of multiple signaling pathways, and leads to cancer progression [24,25]. Previous studies have indicated that EMT is identified as a mechanism resulting in the invasive phenotype of GBM cells [26,27]. TGFβ signaling pathway plays an important role in regulation of EMT, and is activated in high-grade gliomas, leading to poor prognosis [28]. TGFβ activates type I and type II serine-threonine kinase receptors, TbRI and TbRII, leading to the activation of receptor-regulated Smads (R-Smads), Smad2 and Smad3, which further form heterotrimeric complexes with co-Smads and Smad4 [29,30]. The complexes translocate into the nucleus, and regulate EMT target genes through interacting with various transcription factors [30]. Smad2/3, an intracellular signaling molecule, activates different EMT transcription factors [31]. In the present study, we found that the protein level of P-Smad2/3 was inhibited by HCK knockdown in GBM cells, suggesting HCK is involved in EMT via TGFβ/Smad signaling pathway. In addition, we further demonstrated that N-cadherin expression was also decreased in GBM cells with HCK inhibition. N-cadherin, a calcium-dependent single-chain transmembrane glycoprotein, mediates homotypic and heterotypic cell-cell adhesion, playing a critical role in the regulation of the nervous system, brain, heart, skeletal muscles, blood vessels and hematopoietic microenvironment [32]. Furthermore, N-cadherin is a marker of EMT. It is well known that EMT is defined as the decreased expression of the transmembrane protein E-cadherin and the excessive accumulation of mesenchymal markers such as N-cadherin [33]. Previous study has reported that N-cadherin is highly expressed in various cancer, including lung cancer, breast cancer, prostate cancer and squamous cell carcinoma, and abnormal expression of N-cadherin is associated with tumor aggressiveness [32]. In a word, during the EMT process, the mesenchymal markers, such as N-cadherin, are increased [34]. Our results showed HCK knockdown inhibited P-Smad2/3 and N-cadherin expression in GBM cell lines, revealing that HCK inhibition blocks EMT process.

## Conclusion

The present study demonstrated that HCK was highly expressed in tumor tissues from patients with GBM and GBM cell lines. HCK caused an augment of cell viability, proliferation, migration, and tumor growth, and induced cell apoptosis. GSEA showed HCK was closely associated with EMT, and it is further verified by western blotting assay that HCK knockdown decreased the protein levels of P-Smad2/3 and N-cadherin. These results indicate that HCK is involved in GBM progression via mediating EMT process, and may be served as a promising therapeutic target for GBM.

## Supplementary Material

Supplementary Figure S1

## Abbreviations

ATCC: American Type Culture Collection
CCK: Cell Counting Kit
CML: chronic myeloid leukemia
DMEM: Dulbecco's Modified Eagle's Medium
EMT: epithelial mesenchymal transition
ERK: extracellular regulated protein kinases
GBM: glioblastoma
GSEA: gene set enrichment analysis
HCK: hematopoietic cell kinase
HCK-OE: HCK overexpressed
PVDF: polyvinylidene difluoride
qRT-PCR: quantitative reverse transcription polymerase chain reaction
SFK: Src family protein-tyrosine kinase
STAT3: signal transducer and activator of transcription 3
