# Supplementary material for: HCK promotes glioblastoma progression by TGFβ signaling
Source: Biosci Rep. 2020 Jun 17;40(6):BSR20200975. doi: 10.1042/BSR20200975 (PMC7300285; doi:10.1042/BSR20200975)

Supplementary Figure 1: HCK was closely associated with EMT (a) and TGF β signaling (b) in GBM.

Supplementary Figure 1

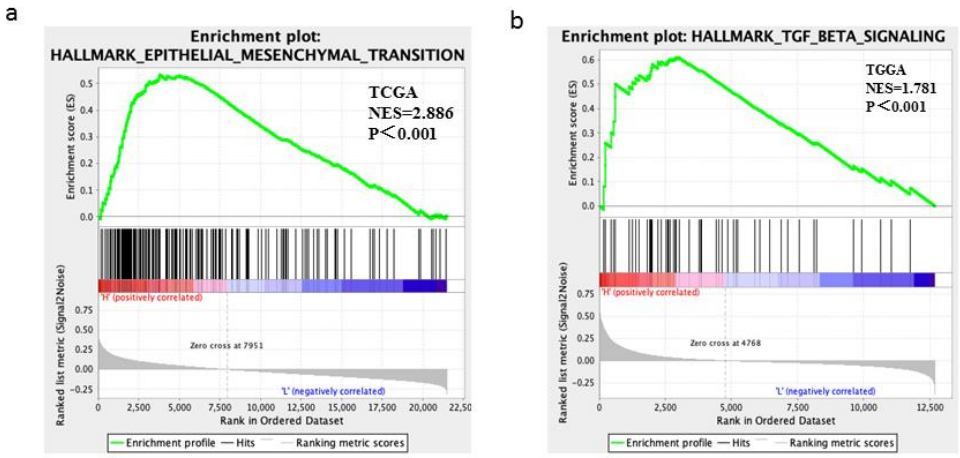

Supplement: Supplementary Figure S1 [file BSR-2020-0975_supp.pdf]
